# Genetic insights of sleep apnea symptomatology and endotypes

**DOI:** 10.1093/sleep/zsaf308

**Published:** 2025-11-13

**Authors:** Hanna M Ollila

**Affiliations:** Institute for Molecular Medicine Finland, Helsinki Institute of Life Science (HiLIFE), University of Helsinki, Helsinki, Finland; Broad Institute of MIT and Harvard, Cambridge, MA, USA; Center for Genomic Medicine, Massachusetts General Hospital, Boston, MA, USA; Department of Anesthesia, Critical Care, and Pain Medicine, Massachusetts General Hospital, Boston, MA, USA

Sleep apnea is one of the most common sleep disorders, affecting over 15% of the population [1, 2]. It is strongly associated with increased cardiovascular morbidity and mortality, making timely detection and treatment critical [3, 4]. Biomarker and genetic studies particularly those focused on obstructive sleep apnea have consistently demonstrated a shared genetic architecture with cardiometabolic diseases, where the same genetic variants contribute to elevated risk for sleep apnea, high body mass index (BMI), and cardiovascular disease [5, 6]. Importantly, additional genetic factors have been identified that influence sleep apnea risk in individuals with low BMI or after adjusting for BMI, highlighting distinct biological pathways [6–9]. However, sleep apnea severity varies across and within diagnostic categories, including in the level of severity of objectively measured and perceived symptoms during wake such as severity of daytime sleepiness. It is likely that different biological mechanisms contribute or cause different clinical manifestations of sleep apnea both in objectively measured parameters and those typically reported by patients.

In recent years, genetics has emerged as a powerful tool to dissect this heterogeneity, not only as a study of the shared architecture between sleep apnea and traits such as BMI and cardiovascular disease, but also providing a lens to understand variation in symptom profiles such as excessive daytime sleepiness [10]. In this issue of *SLEEP*, Nagarajan et al. present a compelling genome-wide interaction study of the apnea-hypopnea index (AHI), a standard measure of sleep apnea severity where the authors use sleepiness as an environment (gene-by-sleepiness interaction) [11]. Their findings build on growing evidence that sleep apnea is not a single entity but a biologically diverse disorder, with daytime sleepiness potentially marking a high-risk subtype with distinct genetic architecture and genetic risk factors.

The genetic risk factors of sleep apnea have been largely studied in relation to BMI. This body of work has helped establish shared risk variants between sleep apnea, obesity, and cardiometabolic disease [5, 6, 9]. However, considerable interindividual variability remains unexplained, including the question why some patients with high BMI have severe sleep apnea while others do not. Similarly, only a subset of individuals experience daytime fatigue, while others feel unaffected. The current study by Nagarajan et al. [11] suggests that sleepiness modifies the genetic architecture of AHI, implicating distinct biological pathways. For example, carriers of *CCDC3* and *MARCHF1* risk variants had a higher AHI value, but only if they reported sleepiness. Such gene by environment (GxE) interactions emphasize that sleepiness may not just reflect symptom burden, but an alternative pathophysiology perhaps one more responsive to inflammation, metabolism, or central nervous system vulnerability.

Overall, our understanding of sleep apnea is moving from a binary diagnosis of sleep apnea toward a complex, quantitative trait that is affected by a polygenic component and environmental risk factors. This view aligns with the broad spectrum of symptoms and clinical heterogeneity seen in clinical practice. Accordingly, by treating AHI as a continuous outcome and incorporating sleepiness as a modifier of genetic effect size, Nagarajan et al. identify several genetic risk loci through common and rare variant analyses [11]. These include genes related to insulin signaling (*MARCHF1*), neuropeptide activity (*NPY1R*, *NPY5R*), thiamine metabolism (*DHTKD1*, *TKTL2*), and immune function with several genes also affecting more than one biological process. Some are novel to the sleep apnea literature, while others have known connections to cardiometabolic diseases. These findings are in line with the growing evidence that sleep apnea is biologically both polygenic and pleiotropic having several genetic risk factors and shared mechanisms between diseases.

A common goal of biomarker science is in its ability to move beyond diagnostic categories and toward biologically defined subgroups. The identification of sleepiness-interacting genetic loci provides potential biomarkers to stratify risk, monitor disease progression, and plan interventions. Particularly promising may be the metabolic pathways observed in the Nagarajan et al. study that may point toward novel therapeutics in the area of sleep apnea [11]. These could include targeting thiamine or resveratrol supplementation in sleepy, high-risk patients or exploring NPYR5 antagonists in those with metabolic dysregulation.

Finally, the integration of sleepiness into genetic analysis exemplifies a broader shift toward understanding complex diseases in the context of the environment. Rather than treating environmental exposures and symptoms as noise, studies like this highlight the value of environmental risk factors in revealing gene function. In the current study, genetic risk factors had a larger effect depending on sleepiness. Such findings challenge the assumption of fixed genetic risk and support a model where genetic liability of sleep apnea is dynamic and influenced by behavior, inflammation, adiposity, and perhaps even additional circadian factors.

Overall, sleep apnea is a complex, multifactorial disorder in which symptoms like daytime sleepiness may provide novel clinically and biologically meaningful subtypes. As Nagarajan et al. demonstrate [11], modeling genetic risk in the context of such symptoms reveals novel loci and pathways offering insights into disease heterogeneity, potential biomarkers, and therapeutic opportunities.
